# Kaahaajat: Finnish Attitudes towards Speeding

**DOI:** 10.3390/ijerph20031995

**Published:** 2023-01-21

**Authors:** Steve O’Hern, Valtteri Vuorio, Amanda N. Stephens

**Affiliations:** 1 Transport Research Centre Verne, Tampere University, 33014 Tampere, Finland; 2 Accident Research Centre, Monash University, Clayton, VIC 3800, Australia

**Keywords:** driver behaviour, speeding, theory of planned behaviour, Finland, road safety

## Abstract

People driving in excess of the posted speed limit (referred to as speeding in English or Kaahaajat in Finnish) is a common road user behaviour. In Finland, between 2000 and 2020, speeding was identified as the key contributing factor in 41% of fatal motor vehicle collisions. This may be because disregarding speed limits on motorways and on residential roads are the most common violations performed by Finnish drivers. This study identifies factors influencing speeding while driving in Finland. In particular, 703 responses from Finnish drivers of the ESRA2 (E-Survey of Road users’ Attitudes) were analysed to understand the theory of planned behaviour (TPB) factors underpinning speeding behaviours in three road environments: inside built-up areas; outside of built-up areas; and on motorways and freeways. Three binary logistic regression analyses were used to understand which elements of TPB were associated with self-reported speeding in each of these environments. Approximately two thirds of participants reported speeding in each of the three road environments. Attitudes and subjective norms were associated with speeding in built-up areas and on motorways or freeways. In addition, perceived behavioural control and age were significantly associated with speeding outside of built-up areas. The findings highlight how a systematic approach is needed to address speeding considering enforcement, engineering, legislation, and education.

## 1. Introduction

People driving in excess of the posted speed limit (Kaahaajat in Finnish), is a common road user behaviour [1,2]. The European Transport Safety Council estimates that between 35% and 75% of vehicle speed observations are higher than the legal speed limit [1]. Excessive and inappropriate speeds are a major road safety problem. Yet even relatively small increases above the posted limit can increase the risk of crash involvement [3,4]. Indeed, speeding is a contributing factor in approximately one third of fatal collisions throughout Europe [1] and a significant number of serious injury crashes [5]. Furthermore, the contribution of speeding in casualty crashes may be underestimated, with the role of speeding in fatal crashes estimated to be as high as 60% when combining data from multiple sources [6].

Vehicle travel speed (“speed”) influences road safety in two ways. First, speed is a direct contributor to kinetic energy, which is converted into the deformation of vehicles, heat, and biomechanical energy in a collision. The more kinetic energy, the more destructive the crash. Speed, therefore, directly influences crash and injury severity [5]. Speed also influences safety by giving road users less time to process information, react, and smaller margins for error when a critical situation occurs. It is widely accepted that excessive and inappropriate speeds for the driving conditions are risk factors for crashes and injury and ceteris paribus, higher speeds are associated with a higher number of crashes [5].

To achieve the United Nations (UN) goal of halving traffic deaths and injuries by 2030 [7], meeting the objectives of Vision Zero, there is a need to address behaviours such as speeding. These objectives are also central to the Finnish road safety strategy.

In Finland, statistics from in-depth investigations of fatal road crashes reported by the Finnish Crash Data Institute “Onnettomuustietoinstituutti” (OTI), show that between 2000 and 2020, speeding was identified as the key contributing factor in 41% of fatal motor vehicle collisions [8], with almost a quarter of drivers involved in these crashes estimated to be exceeding the speed limit by over 30 km/h [8]. When investigating self-reported driver behaviours in Finland, Mesken et al. (2002) [2] found that disregarding the speed limits on motorways and on residential roads were the most common violations performed by Finnish drivers. Mesken et al. identified that speeding violations were committed because drivers want to get to their destinations on time, maintain speed, or because driving fast can be enjoyable for the driver [2]. More recently, when investigating young drivers in Finland, Mattsson replicated the findings of Mesken with a unique sample, while identifying speeding-related items as having the strongest loadings for their rule violation factor [9]. However, these studies focused on the Driver Behaviour Questionnaire and did not consider the underlying reasons as to why some drivers choose to speed. Developing a deeper understanding of the factors that influence a driver’s decisions to speed is imperative to addressing road safety issues. This is particularly important in Finland given both the high prevalence of speeding and the high proportion of crashes where speeding is a contributing factor.

A number of studies have used the theory of planned behaviour to better understand speeding behaviour [10,11,12]. Developed by Ajzen (1991) [13], the TPB is a social psychological model which describes the relationship between socio-cognitive factors and self-reported behaviour. The model stipulates that behaviour can be predicted by a combination of positive attitudes towards the behaviour, perceived behavioural control (PBC) over engaging in the behaviour, and favourable views of the behaviour from others. These factors increase the intention and ultimately engagement in the behaviour. In each case, intention to speed was predicted by more positive attitudes toward the behaviour and greater subjective norms towards it [10,11]. Conner et al. also showed that past behaviour was an important predictor of speeding, thus drivers in Finland who commonly commit speeding violations are more likely to commit these again [12].

Studies using the TPB therefore highlight areas for intervention. Indeed, Stead et al. and Poulter et al. showed that speeding was successfully reduced after interventions targeted the TPB factors [11,14]. These studies, conducted in the UK, indicate that similar interventions would be beneficial to support road safety in Finland. The first step in designing these is to therefore understand whether the TPB model is similarly predictive of speeding behaviour in Finland.

In 2015, the Vias Institute established and conducted the E-Survey of Road Users’ Attitudes (ESRA) [15]. The ESRA aims to collect an international sample of road safety performance data, focusing on road safety culture and self-reported behaviours of road users, including self-reported engagement in speeding while driving and underlying reasons for this behaviour [16]. To this end, the ESRA measures motivation for behaviour using the theory of planned behaviour framework (TPB) [13].

In 2018, the ESRA ran for the second time in 32 countries, with an additional 16 countries surveyed in 2019. Amongst the European nations to participate in ESRA2, over half of the drivers surveyed reported exceeding the speed limit while driving in the 30 days prior to completing the survey [17]. Finnish drivers reported some of the highest prevalence of speeding on motorways, in built up areas, and outside of built-up areas, with the Finnish rates exceeding the averages reported in Europe, Asia, Oceania, North America, and Africa. Similarly, in the 2015 version of the ESRA, Finnish drivers reported the highest rates of speeding with 84% of drivers reporting that they exceeded the speed limit at some stage when driving on motorways in the past 12 months [18].

It is apparent that speeding represents a major road safety issue in Finland with both a high prevalence of self-reported speeding amongst the population and a high proportion of crashes reporting speeding as a key contributing factor. Research conducted elsewhere has shown that the TPB is a promising framework to understand why drivers speed, and design appropriate interventions. However, to date, the TPB has not been applied on a cohort of Finnish drivers. The aim of this research was to use the ESRA2 for the Finnish sample to understand the TPB factors underpinning speeding behaviours reported in the questionnaire.

## 2. Materials and Methods

### 2.1. Procedure

Each country that participates in the ESRA collects a sample of roughly 1000 responses [16]. In Finland, the survey was facilitated by the Finnish Road Safety Council (Liikenneturva). To be eligible for the study, respondents had to be aged 18 or older and reside in Finland. Quotas for the sample were set for age and gender distributions based on the UN statistical division. The geographic location of respondents was also monitored [16].

The online survey took approximately 20 min to complete. The Vias Institute’s protocol was followed for data cleaning and processing [16]. Deidentified data were provided for the analysis. The Finnish National Board on Research Integrity does not require a review by an ethics committee for research based on public and published data, registry and documentary data, or archive data. Notwithstanding, institutional ethics procedures were followed for this research.

A detailed explanation of the ESRA2 methodology is available on the ESRA website (https://www.esranet.eu/, accessed on 7 November 2022) [16].

### 2.2. Materials

The ESRA2 uses the TPB framework to understand motivations behind behaviours of different road users including car drivers [16]. Within the ESRA2, there are a sub-set of questions related to self-declared speeding while driving in built up areas, on motorways and freeways, and outside of built-up areas. Participants were asked; over the last 30 days, how often did they as a car driver speed in each of the three road environments. Responses were recorded on a five-point scale where 1 was “never” and 5 was “almost always”.

The questionnaire also asked respondents about their attitudes towards speeding, their views regarding the acceptability of speeding from a social and personal perspective, opinions of speed enforcement, and their views regarding the risk of speeding while driving. 

### 2.3. Participants

The Finnish sample for the ESRA2 included 994 responses. However, only respondents who held a valid driver’s licence and had driven a car in the 30 days prior to the survey were included in the analysis. This reduced the sample to 703 responses. The remaining 291 responses were excluded from the analysis. Table 1 presents a summary of the respondents’ demographics. Of the included responses, 46.8% were female, and 53.2% were male. Participants’ age was from 18 to 83 (M = 49.9; SD = 17.1). About one-third of the sample lived in urban areas (33.6%) while the remainder lived in semi-urban or rural areas (66.4%).

### 2.4. Analysis

Summary statistics are presented for speeding in the three different road environments. Comparisons are made to the international responses of the ESRA2 survey. Mean and standard deviations are presented for item scores for each TPB factor related to speeding while driving as well as factors related to risk perception, and perceptions of enforcement.

Bivariate associations between age, gender, urbanisation, factors of the TPB, risk perception, and enforcement were measured using Pearson’s and point biserial correlations. Relationship strength followed Cohen’s interpretation of <0.3 weak, 0.30 to 0.50 medium and >0.50 strong relationship [19]. Finally, binary logistic regression was utilised to investigate the dichotomous relationship between drivers that self-reported speeding in each road environment and the associated factors. The analysis was performed using IBM SPSS version 28.

## 3. Results

Participants self-reported speeding in three road environments in ESRA2. Participants were asked ‘Over the last 30 days, how often did you as a car driver …?; drive faster than the speed limit inside built-up areas?, drive faster than the speed limit outside built-up areas (but not on motorways/freeways)?, and drive faster than the speed limit on motorways/freeways?’ Finnish drivers reported some of the highest rates of speeding in the three road environments. Table 2 includes a summary of the Finnish results for the three road environments compared to other Nordic countries and the European, American, Asia/Oceanic, and African averages based on the sample of 1000 responses collected in each country.

Summary statistics for each item related to the TPB (attitudes, subjective norms, and perceived behaviour control), perceptions of enforcement, and risk perception are presented in Table 3. Overall, roughly two-thirds of participants reported speeding in each of the three road environments measured in the questionnaire. However, the rate of engagement was low, with the most common road environment to speed in being motorways and freeways. Generally, drivers had respectful attitudes towards the speed limit, and they did not perceive that obeying the speed limit would cost them time. Participants were neutral regarding how socially acceptable the public found speeding. Similar personal views were held, albeit there was a slightly more positive view towards speeding on motorways, which corresponds with the higher rates of speeding reported in this road environment. Participants tended to not perceive that they had the behavioural control to drive significantly faster than the speed limit or drive fast around sharp corners. There was a slight perception that police may be enforcing speed on a typical drive and there was a general agreement that speed is a contributing factor in crashes involving a car.

Dimension reduction was performed for each factor by averaging scores across the items presented in Table 3. Correlation analysis was performed for each TPB factor, enforcement, risk perception, and participants demographics (Table 4). Overall, relationships between the TPB factors tended to be moderate (ranging from 0.49 to 0.63). Age shared weak negative relationships with self-reported speeding, attitudes, subjective norms, and PBC, indicating that younger drivers tended to also report increased likelihoods of engaging in speeding, more positive attitudes towards speeding, more normalised behaviour in the community and greater perceptions of behavioural control to engage in speeding. There was also a weak relationship between gender, attitudes, and self-reported behaviour. Male drivers tended to report having engaged more in speeding and had more negative attitudes, higher PBC, and higher subjective norms regarding speeding compared to females. Female drivers tended to report a greater perception of risk associated with speeding. Interestingly, there were no relationships between speeding behaviours and geographic location; however, those living in semi-urban and rural areas also tended to feel they were less likely to be checked by police for speeding on a typical journey, albeit this relationship was weak. Moderate to strong relationships were identified between the TPB factors and self-reported speeding. Those who engaged more in speeding also reported a reduced risk perception, and an increased perception of encountering speeding enforcement while driving.

Binary logistic regressions were conducted using self-reported speeding in the three road environments as the dependent variables. The original speeding variables were dichotomized to investigate the differences between participants who reported that they had not engaged in speeding in the past 30 days (i.e., responded 1 to the question) and those that had (i.e., responded 2–5 to the question, as reported in Table 2).

Attitudes (OR = 1.446; 95% CI: 1.203–1.737) and subjective norms (OR = 1.311; 95% CI: 1.219–1.410) were positively associated with speeding in built-up areas, with participants who held positive views towards speeding and felt that the behaviour was accepted amongst the community having higher odds of speeding in built up areas. (Table 5).

When considering speeding on freeways and motorways, attitudes (OR = 1.392, 95%CI 1.152–1.683) and subjective norms (OR = 1.255; 95%CI:1.140–1.317) were again significantly associated with the increased odds of speeding (Table 6).

When driving outside of built-up areas, attitudes (OR = 1.297; 95%CI: 1.062–1.585), and subjective norms (OR = 1.341; 95%CI: 1.237–1.453) were again positive predictors of the behaviour as well as perceived behaviour control (OR = 1.188; 95%CI: 1.007–1.402). Age was also significantly associated with the increased odds of having driven over the speed limit (Table 7).

## 4. Discussion

In Finland, speeding has been identified as the key contributing factor in 41% of fatal motor vehicle collisions [8]. This study investigated the Finnish sample of responses from the ESRA2 survey focusing on self-reported speeding in the three road environments reported in the questionnaire.

Finnish drivers were found to have some of the highest rates of speeding amongst ESRA2 countries. Amongst the three road environments considered in the survey, 78.9% of drivers reported speeding when driving outside of built-up areas, 77.8% of drivers reported speeding on motorways or on freeways, and 72.8% of drivers reported speeding inside built-up areas. The high rates of speeding align with previous research from Finland when investigating self-reported driver behaviours where disregarding the speed limits on motorways and on residential roads were identified as the most common violations performed by Finnish drivers [2].

Compared to other Nordic countries, self-reported rates of speeding were similar on motorways and outside built-up areas; however, the rate of speeding in built-up areas was considerably higher. Finland has one of the better track records for road safety amongst OECD nations [20]. However, Finland has the highest rates of fatalities per population amongst Nordic countries [20]. As such, road safety efforts to target speeding in built-up environments may represent an important intervention. Research by Kloeden et al. [3] found that in 60 km/h urban speed zones, the relative risk of a crash doubles for every additional 5 km/h that vehicles travel over the speed limit. Similarly, Elvik (2008) identified that the number of fatal and serious injury crashes in 60 to 80 km/h speed zones could be reduced by 22% with the elimination of speeding [21]. As such, interventions to reduce speeding in built-up environments could result in significant road safety improvements in Finland.

For interventions to be effective, an understanding of the road users engaging in speeding is required. The findings of this research align with previous studies when comparing bivariate relationships between overall speeding and demographics, and found that young drivers [4,22,23], and males [23,24] were more likely to report engaging in speeding. However, when considering the three specific road environments, no gender differences were identified, and age was only associated with having exceeded the speed limit outside of built-up areas. More surprisingly, older adults were increasingly likely to speed in these environments with each year of age associated with a 2% increase in the odds of having driven over the speed limit outside of built-up environments. The finding suggest that more nuanced classifications of drivers are needed beyond demographics to identify drivers that are at risk of engaging in speeding.

Watson and colleagues proposed that drivers should be classified according to the magnitude and frequency of their speeding. In their study, they found differences in the profile of high- and low-level speeding, both in terms of their age and gender profiles and recommended specific targeted safety strategies related to speed enforcement accordingly [25]. Likewise, Stephens et al. (2017) found that in a representative sample of drivers from Australia, drivers could be classified into different speed behaviour categories [4]. These categories reflected the magnitude of the speed behaviour, from compliant, small exceedances up to 5 km/h over the limit, between 6 and 10 kilometres over the limit, between 11 to 15 kms over the limit, and 16 km over the limit. In line with Watson et al., the demographics in Stephens’ speed categories differed, with younger and male drivers over-represented in the higher speed categories [4]. In addition, those in the higher speed categories were also more likely to have positive attitudes towards speeding behaviour, have friends or family that also speed, and underestimate the risk. Thus, these findings not only align with the data from Finland, demonstrating motivations underlying behaviour, but suggest that different groups of speeders may need to be targeted with different interventions.

Using the TPB, our results build on this by showing that the underlying determinants for speeding differed depending on the road environment. Within each road environment, driver attitudes and subjective norms were associated with self-reported speeding. This provides evidence for potential countermeasures to reduce dangerous behaviour. For example, in their study targeting speeding behaviour using the TPB in the UK, Stead et al. found that advertising campaigns can be effectively used to influence attitudes towards speeding; however, in the same study, they did not identify significant changes amongst subjective norms [11]. Stead suggests that attitudes may be more susceptible to change, compared to subjective norms and PBC when using communication means as they only comprise internal dimensions. As such, interventions could be developed to address attitudes through educational and public awareness campaigns targeting speeding across the three road environments, particularly in built-up environments where Finland falls behind other Nordic countries in terms of driver behaviour. These campaigns could also highlight the risks associated with speeding, which helps raise drivers’ awareness of its contribution to crashes.

When considering speeding outside of built-up environments, the significant factors also included PBC and driver age. In line with Stead’s research, these constructs may be more difficult to influence through public awareness campaigns [11]. To address speeding in these environments, it may be necessary to utilise enforcement strategies. The bivariate analysis identified that there was a generally neutral perception amongst drivers that there was a risk of encountering police enforcement when driving and previous research has shown that speeding is often underpinned by perceptions of enforcement and crash risk [4]. Mackay et al. found that drivers who frequently speed are less likely to perceive that speeding contributes to crashes and are also more likely to hold negative views of enforcement [26]. Finland is one of the few countries in the world to have a “day fine”-based system where fines for speeding over 20 km/h are based on the offender’s personal income. While this system has resulted in some very large speeding fines being issued [27], the findings suggest that Finns do not perceive enforcement as a strong deterrent and that potentially there needs to be an increased effort towards enforcing low-level speeding. This aligns with previous studies which have found that enforcement alone is not an effective measure in reducing aberrant driving behaviours [28,29] and as such, complimentary strategies should also be considered. Notwithstanding, there is scope for further exploration of the issues of enforcement beyond what is covered in the ESRA. The question used in the ESRA asks how likely it is that a car driver would be checked by police; however, it would also be interesting to understand how likely drivers thought it would be that they would be caught speeding and/or receive a fine. This highlights the need for more targeted research looking into the speeding behaviours of drivers beyond what is included in the ESRA questionnaire.

Advanced Driver Assistance Systems (ADAS), such as Intelligent Speed Assist (ISA), may increasingly mitigate speeding behaviours [4]. ISA alerts drivers when they are travelling above the speed limit and has been found to be effective in reducing speed on compliance in car drivers [4]. ISA alongside other ADAS features became compulsory in new vehicles in the EU in 2022 (Regulation (EU) No. 2019/2144) [30]. However, when asked about ISA in the ESRA survey, Finnish drivers held neutral views regarding ISA being installed in new cars, and their mean results were lower than the European average reported in the study [17]. Higher levels of automation may further reduce the risks associated with speeding by reducing the contribution of human errors in crashes [31]. However, these systems rely on drivers adopting the technology and using it correctly. Until the fleet is fully autonomous, other approaches are required to address speeding.

While the ESRA survey offers an understanding into self-reported speeding, there is a need to develop a stronger understanding of the prevalence of drivers who are engaging in speeding, the extent that they are exceeding the speed limit, and the road environments where speeding is most prevalent. Previous research has shown that drivers most often engage in low-level speeding where they exceed the speed limit by up to 5 or 10 km/h [4]. Quantification of the extent of speeding would provide greater insight into the risks associated with the behaviour, beyond what is capable from a self-reported questionnaire and there is a need for complimentary research that quantifies the extent of speeding and the ranges in which speeding is engaged throughout Finland.

There is also a need to develop a comprehensive set of items for assessing the TPB constructs to provide confidence in the construct reliability. There is also a need to further understand the context in which speeding occurs. The ESRA2 data collection was performed in winter, a time when fewer drivers are likely to speed due to the increased workload associated with driving in dark, snowy, and icy conditions which are typical of Finnish roads [32]. Research considering seasonal variation could provide the available insight into enforcement and public awareness campaigns.

Finally, the research is susceptible to biases of self-reported data. Participants may experience recall error when reporting their engagement in speeding. Furthermore, there is the potential that not all participants interpret the Likert scales in the same way. There may also be a desirability bias amongst participants; however, given that the survey was anonymous, this bias should have been minimised.

Notwithstanding, the study increases the understanding of the factors that influence speeding in Finland. The study demonstrates that speeding is a complex issue. A systematic approach is needed to address speeding that uses enforcement, engineering, legislation, and education.

## Figures and Tables

**Table 1:** ESRA2 Finland: Characteristics or car drivers.

Variable		*n* (%)
Gender	Female	329 (46.8)
	Male	374 (53.2)
Age group	18–24	67 (9.5)
	25–34	97 (13.8)
	35–44	106 (15.1)
	45–54	126 (17.9)
	55–64	114 (16.2)
	65+	193 (27.5)
Urbanisation	Urban	236 (33.6)
	Semi-urban or Rural	467 (66.4)

**Table 2:** Proportion of drivers who exceeded the speed limit while driving in the past 30 days.

Country	Inside Built-Up Areas	On Motorways/Freeways	Outside Built-Up Areas
Finland	72.80%	77.80%	78.90%
Denmark	61.80%	74.10%	81.80%
Iceland	73.50%	*	81.40%
Norway	54.10%	79.00%	78.40%
Sweden	53.80%	80.50%	78.50%
America (3)	57.30%	69.90%	64.60%
Europe (24)	56.30%	61.50%	67.50%
Asia Oceania (9)	44.00%	47.90%	47.50%
Africa (12)	41.70%	49.30%	48.80%

* not reported for Iceland, brackets show number of countries in each region, details of the included countries can be found at (https://www.esranet.eu/ accessed on 7 November 2022).

**Table 3:** ESRA speeding item scores and responses to TPB items.

Construct	Item	Mean	SD
Self-reported speeding (1 = never, 5 = almost always)	*Over the last 30 days, how often did you as a car driver*		
	drive faster than the speed limit inside built-up areas?	2.23	1.035
	drive faster than the speed limit outside built-up areas (but not on motorways/freeways)?	2.45	1.090
	drive faster than the speed limit on motorways/freeways?	2.54	1.173
Attitudes (1 = disagree, 5 = agree)	*To what extent do you agree with each of the following statements?*		
	I have to drive fast; otherwise, I have the impression of losing time.	1.46	.822
	Respecting speed limits is boring or dull.	2.04	1.196
Subjective Norms (1 = unacceptable, 5 = acceptable)	*Where you live, how acceptable would most other people say it is for a car driver to*		
	drive faster than the speed limit outside built-up areas (but not on motorways/freeways)?	2.63	1.025
	*How acceptable do you, personally, feel it is for a car driver to*		
	drive faster than the speed limit inside built-up areas?	2.05	1.008
	drive faster than the speed limit outside built-up areas (but not on motorways/freeways)?	2.48	1.109
	drive faster than the speed limit on motorways/freeways?	2.67	1.169
Perceived Behavioural Control (PBC) (1 = disagree, 5 = agree)	*To what extent do you agree with each of the following statements?*		
	I trust myself when I drive significantly faster than the speed limit.	2.07	1.193
	I am able to drive fast through a sharp curve.	1.90	1.117
Risk Perception (1 = never, 6 = almost always)	How often do you think driving faster than the speed limit is the cause of a road crash involving a car?	4.24	1.230
Enforcement (1 = very unlikely, 7 = very likely)	On a typical journey, how likely is it that you as a car driver will be checked by the police for respecting the speed limits?	4.40	1.765

**Table 4:** Bivariate (Pearson/point biserial) correlations.

Factor	1	2	3	4	5	6	7	8
Age (1)	1							
Gender (2)	0.02	1						
Urbanisation (3)	0.05	0.02	1					
Self-reported speeding (4)	−0.09 *	−0.13 **	0.03	1				
Attitudes (5)	−0.15 **	−0.13 **	0.01	0.55 **	1			
Subjective Norms (6)	−0.26 **	−0.16 **	−0.01	0.63 **	0.56 **	1		
Perceived Behavioural Control (7)	−0.16 **	−0.32 **	0.00	0.49 **	0.61 **	0.54 **	1	
Risk Perception (8)	−0.01	0.22 **	−0.03	−0.19 **	−0.23 **	−0.24 **	−0.27 **	1
Enforcement (9)	−0.02	0.02	−0.11 **	0.12 **	0.05	0.13 **	0.05	−0.03

* = *p* < 0.05; ** = *p* < 0.001.

**Table 5:** Factors influencing speeding in built-up areas.

Parameter	B	Std. Error	Sig.	OR	95% CI for OR	
					Lower	Upper
Age	0.010	0.006	0.067	1.010	0.999	1.022
Gender (male)	0.123	0.202	0.543	1.131	0.761	1.680
Urbanisation (urban)	−0.130	0.206	0.527	0.878	0.587	1.314
Attitudes	0.368	0.094	0.000	1.446	1.203	1.737
Subjective Norms	0.271	0.037	0.000	1.311	1.219	1.410
Perceived Behavioural Control	0.040	0.073	0.586	1.040	0.902	1.199
Risk Perception	−0.059	0.084	0.479	0.942	0.799	1.111
Enforcement	−0.018	0.056	0.746	0.982	0.881	1.095
(Constant)	−2.849	0.728	0.000	0.058		

**Table 6:** Factors influencing speeding on freeways and motorways.

Parameter	B	Std. Error	Sig.	OR	95% CI for OR	
					Lower	Upper
Age	0.009	0.006	0.139	1.009	0.997	1.020
Gender (male)	−0.164	0.206	0.427	0.849	0.566	1.272
Urbanisation (urban)	0.131	0.209	0.532	1.139	0.757	1.716
Attitudes	0.331	0.097	0.001	1.392	1.152	1.683
Subjective Norms	0.203	0.037	0.000	1.225	1.140	1.317
Perceived Behavioural Control	0.054	0.076	0.476	1.056	0.910	1.225
Risk Perception	0.040	0.085	0.636	1.041	0.881	1.230
Enforcement	0.038	0.057	0.498	1.039	0.930	1.161
(Constant)	−2.531	0.735	0.001	0.080		

**Table 7:** Factors influencing speeding outside of built-up areas.

Parameter	B	Std. Error	Sig.	OR	95% CI for OR	
					Lower	Upper
Age	0.020	0.006	0.001	1.020	1.008	1.032
Gender (male)	0.009	0.218	0.966	1.009	0.658	1.547
Urbanisation (urban)	0.380	0.219	0.083	1.462	0.951	2.246
Attitudes	0.260	0.102	0.011	1.297	1.062	1.585
Subjective Norms	0.293	0.041	0.000	1.341	1.237	1.453
Perceived Behavioural Control	0.173	0.084	0.041	1.188	1.007	1.402
Risk Perception	0.019	0.090	0.829	1.020	0.855	1.216
Enforcement	−0.013	0.060	0.836	0.988	0.878	1.111
(Constant)	−3.871	0.793	0.000	0.021		

## Data Availability

Data available on request due to restrictions. See https://www.esranet.eu/.
